# Poor consistency in evaluating South African adults with neurogenic dysphagia

**DOI:** 10.4102/sajcd.v64i1.158

**Published:** 2017-01-23

**Authors:** Mckinley Andrews, Mershen Pillay

**Affiliations:** 1 Discipline of Speech-Language Pathology, University of KwaZulu-Natal, South Africa

## Abstract

**Background:**

Speech-language therapists are specifically trained in clinically evaluating swallowing in adults with acute stroke. Incidence of dysphagia following acute stroke is high in South Africa, and health implications can be fatal, making optimal management of this patient population crucial. However, despite training and guidelines for best practice in clinically evaluating swallowing in adults with acute stroke, there are low levels of consistency in these practice patterns.

**Objective:**

The aim was to explore the clinical practice activities of speech-language therapists in the clinical evaluation of swallowing in adults with acute stroke. Practice activities reviewed included the use and consistency of clinical components and resources utilised. Clinical components were the individual elements evaluated in the clinical evaluation of swallowing (e.g. lip seal, vocal quality, etc.)

**Methods:**

The questionnaire used in the study was replicated and adapted from a study increasing content- and criterion-related validity. A narrative literature review determined what practice patterns existed in the clinical evaluation of swallowing in adults. A pilot study was conducted to increase validity and reliability. Purposive sampling was used by sending a self-administered, electronic questionnaire to members of the South African Speech-Language-Hearing Association. Thirty-eight participants took part in the study. Descriptive statistics were used to analyse the data and the small qualitative component was subjected to textual analysis.

**Results:**

There was high frequency of use of 41% of the clinical components in more than 90% of participants (*n* = 38). Less than 50% of participants frequently assessed sensory function and gag reflex and used pulse oximetry, cervical auscultation and indirect laryngoscopy. Approximately a third of participants showed high (30.8%), moderate (35.9%) and poor (33.3%) consistency of practice each. Nurses, food and liquids and medical consumables were used *usually* and *always* by more than 90% of participants.

**Conclusion:**

Infrequent use of clinical components and high variability in clinical practice among speech-language therapists calls for uniform curricula in the clinical evaluation of swallowing at South African universities and for continued professional development post-graduation. Different contexts and patient symptoms contribute towards varied practice; however, there is still a need to improve consistency of practice for quality health care delivery. A research-based policy for the clinical swallowing evaluation for a resource-limited context is also needed.

## Introduction

**FIGURE 1:**
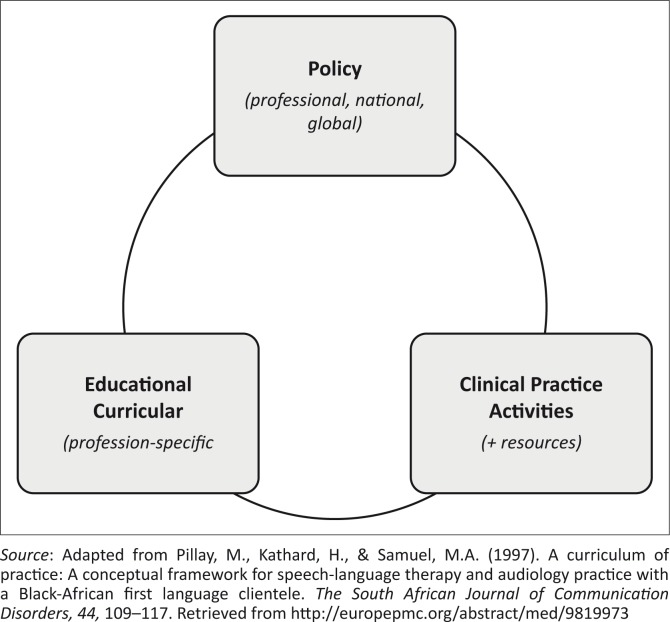
The curriculum of practice.

The focus of this study was on practice patterns used by speech-language therapists in the clinical swallow evaluation of neurogenic dysphagia in adults with acute stroke. Practice patterns are located relative to the ‘curriculum of practice’ (Figure 1) and are defined as consistent activities that have widely led to improved health outcomes (Carnaby & Harenberg, 2013; Mathers-Schmidt & Kurlinski, 2003). This is important for quality of health care and adequate standards of practice (Carnaby & Harenberg, 2013). Practice is also defined as activity related to and influenced by theoretical knowledge, education and training (Pillay, Kathard & Samuel, 1997). Knowledge is moulded by social, political, gender, ethnic, cultural and economic influences (Pillay et al., 1997), and as these influences play large roles in the South African context these will most likely impact speech-language therapists’ practice. Policies are broad practice guidelines based on recent literature, providing the speech-language therapist with general advice on what to do in clinical procedures and how to achieve them (Pillay et al., 1997). Clinical practice activities are those procedures a professional performs and the resources they use so as to manage an adult with neurogenic dysphagia (Pillay et al., 1997). This includes assessing clinical components in the clinical swallow evaluation. Clinical components are the individual elements that make up the clinical swallow evaluation for which the speech-language therapist is responsible for evaluating. Clinical practice is the procedure or protocol that is followed by all health professionals who manage adults with stroke (Davis & Taylor-Vaisey, 1997; Heinemann et al., 2003; Van Peppen, Hendriks, Meeteren, Helders & Kwakkel, 2007). This knowledge–theory relationship gives rise to evidence-based practice which is an effective and high-quality practice that is driven by knowledge from the latest research (American Speech-Language-Hearing Association, 2005) as well as by the speech-language therapist’s competency, experience and preference for practice (Riquelme, 2015). Evidence-based practice is a crucial part of health care delivery as it contributes towards improving health outcomes for those with neurogenic dysphagia due to acute stroke (Straus, Tetroe & Graham, 2009).

A narrative review of the literature on practice patterns of speech-language therapists during the clinical swallow evaluation of adults with neurogenic dysphagia post-acute stroke was performed. A limited amount of literature was available. All 12 studies included in the study showed that most clinical swallow evaluations comprise four main subsections: history, oral motor examination, voice and trial swallows; however, more specific elements still differ among speech-language therapists and have not been officially or extensively outlined by the literature (Riquelme, 2015). Key findings showed that clinical components utilised by more than 90% of speech-language therapists ranged between 24% and 63% across five studies, and the consistency of clinical component utilisation varied between 32% and 58% across three studies. There were infrequent assessments of the gag reflex, sensation and mental status as well as limited use of indirect laryngoscopy, pulse oximetry and cervical auscultation methods. Little information was found on what resources are used during the clinical swallowing evaluation.

Mathers-Schmidt and Kurlinski (2003), Bateman, Leslie and Drinnan (2007) and Pettigrew and O’Toole (2007) observed practice patterns of speech-language therapists in the USA, Ireland and the UK. Infrequent use of clinical components and low levels of consistent use of clinical components in the clinical swallow evaluation were reported in all three studies. Carnaby and Harenberg (2013) also found high variability of practice patterns among speech-language therapists in dysphagia evaluation. Despite receiving both policies guiding clinical practice and relevant education and training, practice still seems to vary internationally among speech-language therapists. Contextual and patient-specific factors will expectedly play a role in differing practice; however, it is expected that higher degrees of clinical components are utilised according to protocols and practice guidelines to ensure a certain standard of optimal health care and evidence-based practice.

The occurrence of stroke and its resulting comorbidities continues to be highly prevalent in South Africa. Stroke is the second highest cause of death in the world and annually claims approximately five million lives (Sajjad et al., 2013). Over four million of these deaths occur in low- and middle-income countries, such as South Africa (Sajjad et al., 2013). Stroke was the fourth leading cause of mortality in South Africa after (1) tuberculosis, (2) influenza and pneumonia and (3) AIDS and was the leading reason for death in adults above the age of 65 years in 2013 (Statistics South Africa, 2014). About 67 000 of South Africa’s population experience a stroke annually (Maredza, Bertram & Tollman, 2015). Dysphagia is a common symptom post-stroke and results in increased mortality and morbidity rates, poor nutrition and dehydration, prolonged disability, and decreased quality of life (González-Fonández, Ottenstein, Atanelov & Christian, 2013b; Guyomard et al., 2009). Up to 50% of adults with stroke are at risk of aspirating and developing pneumonia (Martino et al., 2005). The management of dysphagia post-stroke is therefore important for optimal health care.

South Africa faces a quadruple burden of disease which is aggravated by poverty, high levels of unemployment, socio-economic inequity and an ineffective health system (Mayosi et al., 2012). The four epidemics include HIV and tuberculosis, non-communicable diseases and mental health disorders, deaths related to injury and violence and maternal, neonatal and child mortalities (Mayosi et al., 2012). South African speech-language therapists are therefore often faced with adults with stroke who have additional diseases (Mayosi et al., 2012) that complicate management and prognosis. The South African context can thus be a complex and challenging one for the speech-language therapist.

Dysphagia resulting from acute stroke is managed by speech-language therapists as it is within their scope of practice to conduct a clinical swallow evaluation. The Health Professions Council of South Africa (HPCSA) (2009) stated that the speech-language therapist’s scope of practice is determined by the level of their education, experience and skill. It also stated that services provided must be evidence-based and culturally and linguistically appropriate for the adult with neurogenic dysphagia. The speech-language therapist therefore needs to be up-to-date with clinical swallow evaluation guidelines and recent research in such practices.

Speech-language therapists fulfil this scope of practice by initially using the clinical swallow evaluation, which is a non-instrumental, behavioural assessment procedure involving identifying and interpreting various components of information from the patient, family and various health professionals. The primary goals are to conclude the presence, nature and cause of the dysphagia, and to determine the level of dysfunction, the risk for aspiration and whether nutritional status is adversely affected (Pettigrew & O’Toole, 2007). The result of such an evaluation aids the development of an appropriate swallowing management plan (González-Fonández et al., 2013b). Due to the subjective nature of the clinical swallow evaluation and the grave complications dysphagia may cause it is vital for the speech-language therapist to be informed of the relevant knowledge and skills regarding the clinical swallow evaluation. McCullough et al. (2005) reported that when an adult with neurogenic dysphagia aspirates, silent aspiration occurs half of the time. Silent aspiration also makes the speech-language therapist’s recent knowledge and skills important when performing a clinical swallow evaluation. Silent aspiration occurs when a bolus is aspirated (enters the trachea below the level of the true vocal folds), but no cough reflex is produced as a result (Smith Hammond & Goldstein, 2006).

In South Africa, because the dysphagia evaluation faces many challenges, as discussed below, it is thought that performing a thorough and optimal clinical swallow evaluation can be difficult. Not only is there a dire shortage of trained and skilled health professionals involved in dysphagia management such as doctors and nurses (Department of Health, 2011; George, Quinlan & Reardon, 2009; Mayosi et al., 2012), but they are also unequally distributed between urban and rural areas (Mills et al., 2011). In 2011, South Africa had 7.7 doctors and 40.8 nurses and midwives per 10 000 people in the country (Department of Health, 2011). In sub-Saharan Africa this is exacerbated by HIV, migration of staff to developed countries and a shortage of training institutions to train adequate amounts of health professionals (Mills et al., 2011). Such a shortage of health professionals makes obtaining information about the nature and history of the swallow more challenging, for example obtaining temperature records and a feeding history from a nurse, information from a radiologist regarding the location of brain insult as well as chest status or C-reactive protein information from doctors. There is also a shortage of medical facilities and reduced standard of care due to poor infrastructure and support (Blackwell & Littlejohns, 2010; Mayosi et al., 2012; National Development Plan, 2012). South Africa is culturally and economically diverse. The use of traditional medicine in South Africa is a large trade, and for some living in rural areas it is their only option for health care (Mander, Ntuli, Diederichs & Mavundla, 2007). However, some prefer the treatment from traditional healers (called *sangomas*) to Western medicine, and the speech-language therapist should consider their influence in the clinical swallow evaluation (Blackwell & Littlejohns, 2010). For example traditional healers may have recommended that specific thin fluids be consumed that in fact are not safe to swallow for an adult with neurogenic dysphagia. Knowing such information is valuable during the clinical swallowing evaluation as it contributes information regarding the consistency of liquids the adult is expected to swallow at home. There are 11 official languages in South Africa which often leads to difficulty in communicating effectively with the adult with stroke and their caregivers (Blackwell & Littlejohns, 2010). This makes identifying accurate swallowing history and background information from the caregivers and the adult with neurogenic dysphagia problematic. The adult with neurogenic dysphagia’s ability to follow the speech-language therapist’s instructions during the evaluation also gets adversely affected by such language barriers. The speech-language therapist must consider all these influential factors discussed during the clinical swallow evaluation in the South African context.

There was a need to investigate the protocol that South African speech-language therapists follow during the clinical swallow evaluation of adults with neurogenic dysphagia post-acute stroke, as currently such practice is unknown. Due to the life-threatening nature of dysphagia and the various challenges encountered in the South African context, it is important to explore what practice currently exists and whether it is of adequate standard to ensure safe and optimal management of those who have suffered acute stroke and resulting in dysphagia. The results of this study will ultimately contribute towards a uniform education and training curriculum for the clinical swallow evaluation among South African universities as well as the development of uniform guidelines and policies for such practices. Any poor adherence to existing practice guidelines and clinical component utilisation may contribute towards motivating for uniform curricula. By providing uniform curricula, the quality of training is thought to improve, or at least generalise better clinical practice by increasing levels of preparedness for evaluating dysphagia (Singh et al., 2015).

### Aims and objectives

The aim of the study was to explore the practice patterns that South African speech-language therapists follow in the clinical swallowing evaluation of adults with neurogenic dysphagia due to acute stroke. Objectives included exploring practice patterns in terms of:

- the frequency of clinical component utilisation,
- the consistency of clinical component utilisation,
- the frequency of resources used and
- the factors contributing towards infrequent use of resources.

## Research methodology and design

A survey design was used for the study and exploratory and descriptive designs were also incorporated. A survey design is considered a valuable method of obtaining original data to describe a large population that cannot be observed directly, or are difficult to observe (Leedy & Ormrod, 2013). The study explored and obtained information and more understanding about practice patterns as they have not yet been fully researched (Bless, Higson-Smith & Kagee, 2006).

The researcher performed a narrative review of the literature to inform the adaptation of a questionnaire from a study by Bateman et al. (2007). A narrative review is a thorough review of published literature on a specific topic. It has been known to consist of relatively unsystematic methods, but is a good summary of up-to-date information (Green, Johnson & Adams, 2006). The study was replicated and based on a questionnaire taken and adapted from a study by Bateman et al. (2007), and thus all its clinical components were replicated and included in the current study. Further modifications from a pilot study were applied to the questionnaire to improve its content and layout and overall feasibility (Table 1). Eight participants returned a feedback sheet electronically reporting questionnaire adaptations. Participants had 2 weeks over August and September 2015 to participate in the pilot study. The South African Speech-Language-Hearing Association (SASLHA) recruited participants by e-mail and via the social media website Facebook^®^. Participants accessed the self-administered, electronic questionnaire on the web survey development company website Survey Monkey^®^ over a time frame of 4 weeks in October and November 2015.

**TABLE 1:** Description and rationale for the pilot study adaptations to the questionnaire.

Participant code allocation	Original questionnaire item	Participant response	Adapted questionnaire item
2, 4, 5, 9	-	No difficulties or changes to be made.	-
3, 6	Question 10: How often do you refer to the following health professionals when carrying out a clinical swallowing evaluation?	Change ‘refer to’ to ‘consult with’ or ‘liaise with’, specifically regarding nurses.	Question 16: How often do you refer to, *or consult with*, the following health professionals when carrying out a clinical swallowing evaluation?
6	Questions 2.10 and 2.11: How often do you include assessing *language* abilities in the clinical evaluation of swallowing of adults with strokes? How often do you include assessing *mental* abilities in the clinical evaluation of swallowing of adults with strokes?	Difficulty distinguishing between assessing ‘language abilities’ and ‘mental abilities’.	Questions 7.10 and 7.11: How often do you include assessing language abilities or *cognitive communication abilities* in the clinical evaluation of swallowing of adults with strokes?	How often do you include assessing *mental status* in the clinical evaluation of swallowing of adults with strokes?
6	General instruction: Circle the number that corresponds with your answer.	Exchange the term ‘circle the number’ to ‘click on the number’ due to the electronic nature of the study.	*General instruction: Click on* the number that corresponds with your answer.
6	Question 15: In a typical month, approximately how many adults with strokes do you clinically evaluate for swallowing? *Please provide a numerical answer. Answer:* ______	Consider giving a multiple choice answer as it is difficult to guess a single number of adult with strokes seen in a month. Perhaps provide a choice of numerical ranges as an answer.	Question 4: In a typical month, approximately how many adults with strokes do you clinically evaluate for swallowing? *Please select only one answer.* Answer: 1. 1–20; 2. 21–40; 3. 41–60; 4. 61–80; 5. > 80.
6	Question 5.10: How often do you include *each* of the clinical components in the clinical evaluation of swallowingof adults with strokes? Answer: Assessment of aspiration/opinion of airway safety and respiratory coordination	Suggest adding ‘potential aspiration’ or ‘penetration’. The term ‘opinion of airway safety’ was decided upon to cover all of these terms implicating airway safety.	Question 10.12: How often do you include *each* of the clinical components in the clinical evaluation of swallowingof adults with strokes? Answer: Assessment and *overall opinion of airway safety*.
6	Numerous questions: ‘…evaluation of swallowing of adults with strokes.’	Change ‘*of* adults with strokes’ to ‘*in* adults with strokes’.	Did not change as the original is grammatically sound.
6	Question 14: What is your current job setting? Please indicate approximately how much of your time you spend at each location.	Change ‘spend’ to ‘spent’ because job settings have changed in the past.	Question 3: Did not change as the question regards the participant’s *current* job setting and not time spent in previous job settings.
6, 8	Question 7: What, for you, is the most predictive of aspiration in your clinical swallowing evaluation?	Change the layout of the question to allow for multiple answers (technical difficulty). The wording of the question is ambiguous when asking for one most predictive factor but then allowing for multiple answers.	Question 11: What, for you, *is/are* the most predictive of aspiration in your clinical swallowing evaluation?
7	Question 3: How often do you include *each* of the clinical components in the clinical evaluation ofswallowingof adults with strokes?	It is not always possible to assess speech and language abilities in adults with strokes when his/her condition is severe and resulting aphasiaand/or motor speech disorders are severe. Recommend saying something along thelines of ‘…in those patients that can/cannotcommunicate’.	Question 8: How often do you include *each* of the clinical components in the clinical evaluation ofswallowingof adults with strokes? ‘*This applies when the adult with a stroke is both able to communicate and/or vocalise, or unable to.*’
Researcher	Question 14: What is your current job setting? Please indicate approximately how much of your time you spend at each location. One of the answers: Private Clinic.	Changed ‘Private Clinic’ to ‘Private Rehabilitation Facility’ as the South African participant preferred this term. The word ‘current’ was also highlighted as there was confusion regarding past *v* current job settings. Also emphasised that the allocation of time at each venue needed to make sense, as participants struggled with this.	Question 3: What is your ***current*** job setting? Please indicate approximately how much of your time you spend at each location *you work_at*, bearing in mind it needs to make sense e.g. do not click on *always* for two different venues. One of the answers: Private Rehabilitation Facility.
Researcher	Question 8: How often do you use the following resources when carrying out a clinical swallowing evaluation? Answer: Non-medical resources and Medical resources.	Changed the answer options to include more defined categories with an example for each.	Questions 13, 14 and 16: Answer: Medical: Consumables (e.g. gloves/tongue depressors), Medical Instruments (e.g. stethoscope/oximeter) and Other. Non-medical: Food and liquid items (e.g. mashed potato/water), Eating Utensils (e.g. crockeries/utensils), Food and liquid modifying agents (e.g. food thickeners) and Other.
Researcher	Frequency answers: 0. Never 1. Sometimes 2. Half of the time 3. Mostly 4. Always Or: 1. Never 2. Seldom 3.Half the time 4. Usually 5. Always.	Changed all frequency answers to be the same labels and codes, aiding the raw data capturing process by decreasing confusion and time taken.	Frequency answers: 1. Never 2. Seldom 3. Half the time 4. Usually 5. Always.
Researcher	Question 8: How often do you use the following resources when carrying out a clinical swallowing evaluation? One answer: Other (please specify which other resource you use and how often you use this resource).	Highlighted and capitalised the word ‘and’ to highlight the fact that upon choosing this answer (‘other’) two instructions needed to be completed, not just one.	Questions 13, 14 and 16: How often do you use the following resources when carrying out a clinical swallowing evaluation? One answer: Other (please specify which other resource you use *and* how often you use this resource).
Researcher	Question 6: How often do you include each of the clinical components in the clinical evaluation of swallowing of adults with strokes?	Additional instrumentation was described as ‘methods’ instead of ‘clinical components’ as instrumentation refers to *how* and not *what*.	Queston 12: How often do you include each of the *methods* listed below in the clinical evaluation of swallowing of adults with strokes?
Researcher	Question 4: How often do you includeassessing gag reflex and coughing in the clinical evaluation of swallowing of adults with strokes?	Moved these clinical components from the ‘OralPeripheral Motor Examination’ to the ‘Swallowing Function’ subsection.	Question 10: How often do you includeassessing gag reflex and coughing in the clinical evaluation of swallowing of adults with strokes?
Researcher	-	Ensured all of these subsections had an ‘Other’ option.	Questions 7–10, 12: Other: (Please provide any other clinical components you may evaluate in this subsection):

Participants were selected via purposive sampling. Only people with specific knowledge and skills were invited to take part, with the purpose of being able to provide data competently for the study (Leedy & Ormrod, 2013). Training with regularly working with adults with neurogenic dysphagia enables one to be proficient in assessing this population and thus to provide valid data for the study (HPCSA, 2009). Participants were practitioners who had a speech-language therapy degree in 2014 or earlier. The participants were currently working with adults with neurogenic dysphagia post-acute stroke in South Africa. These participants, therefore, had sufficient and recent experience and knowledge with this population (HPCSA, 2009). A total of 38 participants completed the electronic questionnaire. Babbie and Mouton (2001) state that 5–25 participants in the interpretive paradigm is a sufficient sample size. Most of the participants in the study (*n* = 17; 44.7%) had between 1 and 5 years of experience both as a speech-language therapist and working with adults with neurogenic dysphagia, and 23.6% (*n* = 9) of the participants were completing their community service year (first year of work) (Figure 2).

**FIGURE 2:**
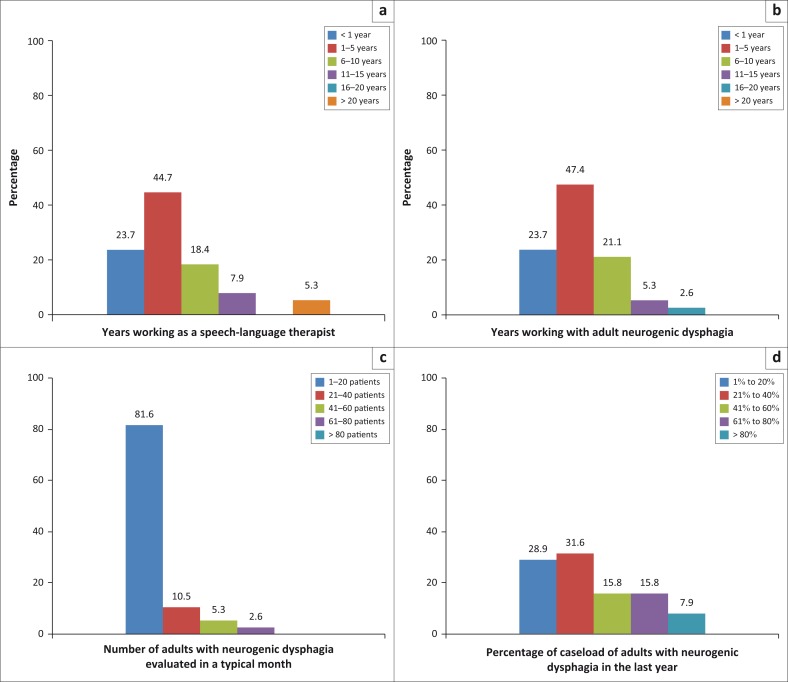
Biographical information of participants (*n* = 38): (a) Years working as a speech-language therapist; (b) Years working with adult neurogenic dysphagia; (c) Number of adults with neurogenic dysphagia evaluated in a typical month; and (d) Percentage of caseload of adults with neurogenic dysphagia in the last year.

Descriptive statistics were used to analyse quantitative data. Raw data were converted into numerical data via coding (Babbie & Mouton, 2001). The frequency of clinical component and resource usage was determined by converting the frequency of codes into percentages (Babbie & Mouton, 2001). The consistency of clinical component use was calculated as how many clinical components were used with the same frequency across participants (Bateman et al., 2007). Those clinical components used with the same frequency by more than 75% of participants were grouped as highly consistent, those between 50% and 75% as moderately consistent and those below 50% as inconsistent (Bateman et al., 2007). The small qualitative component was subjected to textual analysis, where themes were identified in the text and coded into numerical values (Leedy & Ormrod, 2013). The frequency of occurrence of themes was identified by counting how many times each code was recorded, and the themes were discussed (Leedy & Ormrod, 2013).

## Results

### Clinical components in the clinical swallow evaluation

#### The frequency of use of clinical components by participants

Forty-one per cent of the clinical components (16/39) were *usually* or *always* used by more than 90% of the participants (*n = 38*). These included the following: medical history (100%); language abilities or cognitive communication abilities (100%); overall oral efficiency (100%); vocal quality pre- and post-swallow (100%); oral residue (100%); overall opinion of airway safety (100%); medical status (97.4%); variety of bolus types (97.4%); lip seal (97.3%); oral structures, muscles and functioning (97.3%); vocal quality (94.8%); nutritional status (94.7%); laryngeal elevation (94.7%); saliva control/management (94.7%); respiratory status (92.1%); and patient interview/perception of the problem (92.1%). Forty-six per cent of clinical components (18/39) were *usually* or *always* utilised by a range of 50–90% of the participants, and 12.8% (5/39) were *usually* or *always* utilised by less than 50% (0% to 31.6%) of the participants.

#### The consistency of use of clinical components by participants

**FIGURE 3:**
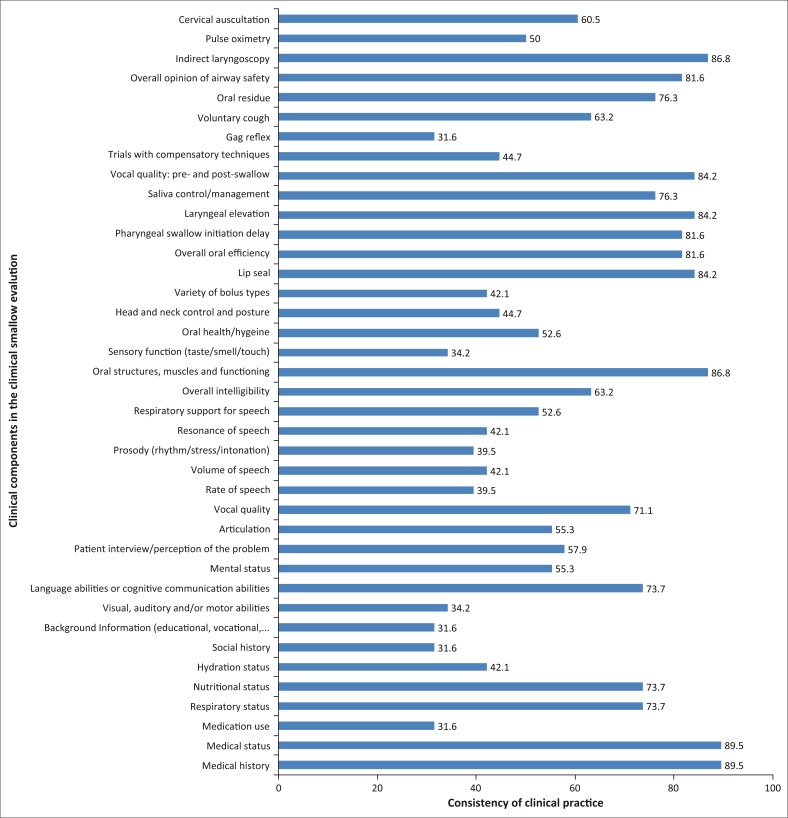
Consistency of clinical component use.

There was a high consistency of clinical practice among participants for 12 out of 39 clinical components (30.8%) (Figure 3). These clinical components included the following: medical history (89.5%); medical status (89.5%); oral structures, muscles and functioning (86.8%); indirect laryngoscopy (86.8%); lip seal (84.2%); laryngeal elevation (84.2); vocal quality pre- and post-swallow (84.2); overall oral efficiency (81.6%); pharyngeal swallow initiation delay (81.6%); overall opinion of airway safety (81.6%); saliva control/management (76.3%); and oral residue (76.3%). Participants showed a high consistency of practice by *always* using these clinical components, with the exception of indirect laryngoscopy, which was *never* used by more than 75% of the participants. Although these clinical components were used highly consistently, no clinical components reached more than 90% consistency.

There were high rates of variability of clinical practice among the remaining participants (69.2%). Clinical practice was moderately consistent for 35.9% of the clinical components (14/39) and inconsistent for the remaining 33.3% (13/39). These included evaluating head and neck control and posture (44.7%); trials with compensatory techniques (44.7%); various features of speech (39–42%); visual, auditory and/or motor abilities (34.2%); and sensory function (34.2%). The clinical components used least consistently included medication use, social history, background information and gag reflex (31.6%).

### Resources in the clinical swallowing evaluation

#### Physical resources

On average 37.9% of responses showed that physical resources (both medical and non-medical) were used with low frequencies in the clinical swallow evaluation (Table 2). Physical resources included consumables, medical instruments, food and liquid items, eating utensils and food and liquid modifying agents. More than half of the responses (57.3%) indicated limited access to physical resources such as medical instruments, adaptive eating utensils and food and liquid modifying agents. Other reasons for using physical resources infrequently included patient-specific needs, theft and limited time, limited staff and limited funding. Also, it was mentioned that other health professionals use certain resources instead; for example, occupational therapists issue adapted spoons.

**TABLE 2:** Frequency and consistency of clinical components and resources utilised in the clinical swallow assessment by participants.

Consistency	Never		Seldom		Half the time		Usually		Always		Total†
	*N*	%	*N*	%	*N*	%	*N*	%	*N*	%	
hc							4	10.5	34	89.5	38
hc					1	2.6	3	7.9	34	89.5	38
ic			6	15.8	10	26.3	12	31.6	10	26.3	38
mc			2	5.3	1	2.6	7	18.4	28	73.7	38
mc	1	2.6			1	2.6	8	21.1	28	73.7	38
ic			1	2.6	7	18.4	14	36.8	16	42.1	38
ic	1	2.6	6	15.8	9	23.7	10	26.3	12	31.6	38
ic	3	7.9	5	13.2	8	21.1	10	26.3	12	31.6	38
ic			8	21.1	9	23.7	13	34.2	8	21.1	38
mc							10	26.3	28	73.7	38
mc	2	5.3	3	7.9	2	5.3	21	55.3	10	26.3	38
mc			1	2.6	2	5.3	13	34.2	22	57.9	38
mc	1	2.6	2	5.3	3	7.9	11	28.9	21	55.3	38
mc					2	5.3	9	23.7	27	71.1	38
ic			7	18.4	6	15.8	10	26.3	15	39.5	38
ic	2	5.3	3	7.9	6	15.8	1	28.9	16	42.1	38
ic	2	5.3	5	13.2	9	23.7	7	18.4	15	39.5	38
ic	3	7.9	7	18.4	4	10.5	8	21.1	16	42.1	38
mc	2	5.3	2	5.3	5	13.2	9	23.7	20	52.6	38
mc	1	2.6	1	2.6	2	5.3	10	26.3	24	63.2	38
hc			1	2.6			4	10.5	33	86.8	38
ic	2	5.3	11	28.9	13	34.2	7	18.4	5	13.2	38
mc			4	10.5	3	7.9	11	28.9	20	52.6	38
ic			1	2.6	4	10.5	17	44.7	16	42.1	38
mc					1	2.6	13	34.2	24	63.2	38
hc					1	2.6	5	13.2	32	84.2	38
hc							7	18.4	31	81.6	38
hc			3	7.9	1	2.6	3	7.9	31	81.6	38
hc			2	5.3			4	10.5	32	84.2	38
hc					2	5.3	7	18.4	29	76.3	38
hc							6	15.8	32	84.2	38
ic			2	5.3	7	18.4	12	31.6	17	44.7	38
ic	7	18.4	12	31.6	12	31.6	4	10.5	3	7.9	38
mc			1	2.6	6	15.8	7	18.4	24	63.2	38
hc							9	23.7	29	76.3	38
hc							7	18.4	31	81.6	38
hc	33	86.8	4	10.5	1	2.6					38
mc	19	50	5	13.2	7	18.4	5	13.2	2	5.3	38
mc	23	60.5	8	21.1	4	10.5	1	2.6	2	5.3	38

† *n* = 38.

**Medical resources:** More than 90% of participants used consumable resources (e.g. gloves, tongue depressors) *usually* and *always* in the clinical swallow evaluation; however, over half (57.9%) of participants *never* and *seldom* used medical instruments such as stethoscopes and pulse oximeters. Fourteen per cent of responses conveyed that little or no training with medical instruments was received, nor were there opportunities for experience with them. Participants expressed a desire for such training as well as for mentoring from experienced clinicians specifically as they felt unprepared and uncomfortable in the work place.

**Non-medical resources:** More than 90% of participants used food and liquid items *usually* and *always* in clinical swallow evaluation, but only 36.9% *usually* and *always* used eating utensils. This may have been because examples given to participants were of adaptive eating utensils (e.g. dysphagia cups) and not typical ones (e.g. spoons and forks). Participants may therefore have reported on the use of only adaptive eating utensils, not eating utensils in general, including typical eating utensils. Just over half of participants (55.5%) *usually* and *always* used food and liquid modifying agents in the clinical swallow evaluation.

#### Human resources

Over 90% of participants consulted with nurses *usually* and *always*, and 86.8% of participants consulted with doctors and allied health professionals like physiotherapists and occupational therapists *usually* and *always* in the clinical swallow evaluation. It was reported that nurses were required for their knowledge regarding the patient’s feeding practices. Occupational therapists and physiotherapists were consulted when evaluating head and neck control, positioning and visual and motor abilities. Physiotherapists also provided information regarding the lungs while occupational therapists assisted with adaptive eating utensils.

Other human resources reported to be in the clinical swallow evaluation were ear, nose and throat specialists, neurologists, social workers, caregivers or family members and dieticians who confirmed whether the patient was malnourished and assisted with food and liquid consistency modifications. Three participants also reported the use of a medical rehabilitation team. Eleven per cent of responses showed that human resources like doctors and nurses were used with low frequencies in the clinical swallow evaluation. Participants reported that human resources were simply not available.

In summary, nurses, food and liquids and medical consumables were used *usually* and *always* by more than 90% of participants. Almost a third (29.1%) of responses showed infrequent (*never*, *seldom* and *half the time*) use of all resources on average (Table 2).

## Discussion

### Clinical components in the clinical swallow evaluation

#### The frequency of use of clinical components by participants

All those clinical components used frequently (41%) in the clinical swallow examination indicated evidence-based practice by participants, as these clinical components are all supported by the literature, are included in policies and in training and thus it is expected that they are frequently included (American Speech-Language-Hearing Association, 2004; Bateman et al., 2007). The remaining 23 clinical components (59%) were used infrequently and are discussed here.

Fifty-eight per cent of participants identified medication use *usually* and *always*. This might have been due to poor education and training after graduation, or challenging circumstances in which to obtain such information. This remains, however, important information to identify in the clinical swallow evaluation as many types of medication can adversely influence dysphagia, such as anti-depressants, medication for blood pressure and for nausea, which can all cause xerostomia (Balzer, 2000). These may need to be treated after receiving a stroke. Any medicine that depresses the central nervous system can limit sensation, awareness and voluntary muscle control (Balzer, 2000). Even the act of swallowing medication can be a hazard in itself (Schiele et al., 2015). It is important that the speech-language therapist not only identify which medication has been prescribed but also in which form the adult with neurogenic dysphagia is consuming it.

General background information (education, vocational, socio-economic and cultural information) and social history were identified by 57.9% of participants *usually* and *always*. This important information is to aid decisions regarding what is assessed in the clinical swallow evaluation and how to go about planning management. One participant mentioned the importance of considering modified diets in accordance with cultural preferences. It is the speech-language therapist’s responsibility to conduct practice that is socially and culturally sensitive, especially in South Africa where the population is culturally and linguistically diverse. Considering culture and diversity consists of more than race and ethnicity and is often overlooked. It includes considering language, religion, customs, values, tastes, lifestyle, education, profession and age, among others. This reduces bias, endorses cultural understanding in practice and puts the adult at ease and increases compliance with the speech-language therapist (Riquelme, 2013). It is also important to understand the adult’s beliefs with regard to matters such as death and certain medical procedures, such as nasogastric tube or percutaneous endoscopic gastrostomy insertion. Other allied health professionals, such as physiotherapists and occupational therapists, are also expected to consider cultural differences in their practice (Riquelme, 2007). Difficulty in communicating, difference in language and/or poor availability of caregivers who can provide background information are often reasons for not assessing this information. Limited and unreliable public transport services in South Africa or the inability of the caregivers to afford transport are often reasons for their inability to get to hospital. Riquelme (2007) recommends using a professional interpreter for language barriers because colleagues or family members may not disclose complete information due to cultural privacy beliefs and credibility of the message may be lost. Such human resources are often not available in South Africa posing a challenge for the speech-language therapist to adequately obtain accurate information in the clinical swallow evaluation.

Vocal prosody was evaluated by 57.9% of speech-language therapists *usually* and *always*. It provides information on the ability of the patient to control aspects such as intonation, rhythm and stress in their speech and can give clues as to whether dysarthria is present or not and which type of dysarthria it may be. The identification of dysarthria aids decisions regarding the presence of aspiration and gives clues to the nature of dysphagia as it can be predictive of aspiration (Schroeder, Daniels, McClain, Corey & Foundas, 2006). It was perhaps not assessed as much because other speech characteristics provide them with more information regarding dysarthria or aspiration. There may also be poor education and training after graduation. Some participants mentioned that a speech function assessment was performed informally, but was not always completed if the patient was medically unstable or due to time constraints where assessing swallowing function took priority.

Fifty-five per cent of participants identified visual, auditory and/or motor abilities frequently. These give the speech-language therapist an idea of the patient’s abilities and limitations. Motor difficulties can affect the patient’s oral musculature and movements and goes hand-in-hand with safe head and trunk positioning for swallowing (American Speech-Language-Hearing Association, 2004; González-Fonández et al., 2013a). Appropriate positioning is an integral factor in terms of safe swallowing. Vision can affect feeding abilities and the ability to hear can affect the following of instructions.

Given the key role that sensation plays in dysphagia, it is concerning that sensation was often evaluated by only 31.6% of participants. Mathers-Schmidt and Kurlinski (2003), Bateman et al. (2007) and Pettigrew and O’Toole (2007) reported higher frequencies of utilisation of this clinical component, which were 74.2%, 56% and 76%, respectively. Sensory feedback helps the triggering of the swallow, chewing and salivary flow and is critical for effective swallowing (Rogers & Arvedson, 2005). Reasons for such poor utilisation of sensation in the clinical swallow evaluation are possibly due to limited resources such as sour, sweet and bitter bolus variations or varying temperature boluses. There are also no clear guidelines or satisfactory measurement techniques for interpretation of oral sensitivity testing (Pettigrew & O’Toole, 2007). It is also interesting to note that South African policies and guidelines do not delineate assessing sensory function in the clinical swallow evaluation. It may also be that speech-language therapists have forgotten how important sensation is in the swallowing process, indicating how continued education after graduating is so important. It was noted that the more experienced speech-language therapists were more likely to evaluate sensation than those currently completing their community service. This is surprising as those speech-language therapists currently completing their community service have recently studied and thus have knowledge and theory that is more up-to-date. This highlights the importance of both clinical experience and continued professional education.

Only 18.5% of participants *usually* and *always* used pulse oximetry methods, and as many as 63.2% of participants *never* and *seldom* used it. The reliability of using pulse oximetry to detect aspiration in the clinical swallow evaluation has received conflicting support in the literature (Chong, Lieu, Sitoh, Meng & Leow, 2003; Ramsey et al., 2003), and thus it seems speech-language therapists decide for themselves whether it should be utilised (Bateman et al., 2007). This may indicate that speech-language therapists are acknowledging the varying support in the literature and using it according to their judgement in the clinical swallow evaluation, thus perhaps successfully translating knowledge into practice. Other reasons for poor utilisation included poor training and poor availability of pulse oximeters (Blackwell & Littlejohns, 2010) to monitor the oxygen saturation levels in arterial blood during the act of swallowing.

As many as 50% of participants *never* or *seldom* utilised gag reflex testing, and only 18.4% of participants assessed it frequently. There is varying clinical usefulness of this clinical component in the literature, and thus it seems speech-language therapists are using it as they see fit (McCullough et al., 2005; Oliveira et al., 2015). Other reasons for poor use were that it was uncomfortable for the patient and that the doctor assessed it. Only 7.9% of participants *usually* and *always* used cervical auscultation procedures, while 81.6% participants *never* and *seldom* used it. This was mainly due to poor training and limited availability of medical equipment. There are conflicting results of the reliability of such a method in the literature, but cervical auscultation has been shown to have generally good input towards the overall picture of the patient’s swallowing ability when used in conjunction with other information obtained from the clinical swallow evaluation (Lagarde, Kamalski & Van den Engel-Hoek, 2015; Ramsey et al., 2003).

Indirect laryngoscopy was the only method that was never (0%) used *usually* or *always*. As many as 97.3% of participants *never* and *seldom* used it. It is evident that the use of this procedure is out of date, as there is very little supporting literature or recent research available on its relevance in the clinical swallow evaluation (Ponka & Baddar, 2013). The indirect laryngoscopy procedure observes the vocal folds at rest and during phonation and is therefore physically irrelevant as it does not provide information on vocal fold competence during swallowing (Ponka & Baddar, 2013). It is therefore expected that few speech-language therapists would be trained to perform such a procedure and those who were trained would perhaps not use it. This procedure is also most often performed by the ear, nose and throat specialist. The poor availability and cost of laryngeal mirrors and time constraints may also limit the frequency of its use. Refer to methodology with regard to reasoning for including this clinical component. It is also valuable to observe evidence-based practice among participants in this study by the fact that this method is not utilised.

The majority of participants (89.4%) *usually* and *always* carried out a clinical swallow evaluation before an instrumental evaluation. The remaining 10.6% of participants reported doing this less frequently. The clinical swallow evaluation provides important information regarding the patient’s oral motor and sensory functioning, ability to follow instructions and the nature of the swallow and it also provides a natural setting for eating and drinking. The clinical swallow evaluation is important for first considering the effectiveness of various postural swallowing techniques and adaptive feeding measures in preventing aspiration. Their effectiveness is confirmed with an instrumental evaluation (González-Fonández et al., 2013b). Omission of the clinical swallow evaluation could be due to doctors or radiographers summoning the speech-language therapist to the instrumental evaluation without the knowledge of an initial clinical swallow evaluation or limited human resources, resulting in less time to perform both a clinical swallow evaluation *and* an instrumental evaluation for every adult with a stroke.

Although more than 90% of participants displayed good clinical practice by frequently using 41% of clinical components in the clinical swallow evaluation, the remaining 59% of clinical components were used infrequently. Despite receiving education, training and policies guiding practice it is understandable that frequency of clinical component utilisation would differ due to: (1) patient-specific requirements and the nature of the clinical swallow evaluation, which is applicable anywhere around the world, (2) the South African context where varying policies are established and (3) where resource availability is limited. Each adult with neurogenic dysphagia post-acute stroke presents with varying difficulties and capabilities, thus requiring individual needs and adaptations in the clinical swallow evaluation. It is the speech-language therapist’s duty to use his or her discretion and tailor the clinical swallow evaluation individually to the needs of the adult with stroke and include or exclude clinical components as he or she sees appropriate (American Speech-Language-Hearing Association, 2004). The clinical swallow evaluation is thus exploratory in nature and is susceptible to change according to the speech-language therapist’s judgement (SASLHA, 2011).

Scope of practice and policies with guidelines for practice set by the HPCSA (2009) and SASLHA (2011) are vague and incomplete. Clinical components that are supported by the literature and should be included in the clinical swallow evaluation are omitted, for example assessing sensation of oral motor structures. Similar policies of international standards such as the American Speech-Language-Hearing Association (2004) or the Speech Pathology Association of Australia Ltd (2012) provide in-depth and complete guidelines and scope of practice detailing all clinical components that should be used in the clinical swallow evaluation. Speech-language therapists in South Africa do not all have access to policies from SASLHA (2011) and some do not know that the HPCSA’s (2009) guidelines for scope of practice exist. Speech-language therapists tend to rely on training from university, Continued Professional Development events and advice from colleagues with regard to what the clinical swallow evaluation should include, instead of adhering to guidelines for practice (Modi & Ross, 2000).

Another reason why the clinical swallow evaluation is likely to vary among speech-language therapists is due to the South African context where availability of resources is limited. Approximately three-quarters of African countries, including South Africa, receive the lowest proportion of government funding for health care (29.5%) compared with high-income countries (42%; George et al., 2009). Low- and middle-income countries have a greater lack of resources and poor access to health care compared with high-income countries. Given the lower socio-economic status and the massive economic burden due to stroke in South Africa there is a shortage of trained and skilled health professionals and a shortage of medical facilities and equipment for stroke (Mills et al., 2011).

#### The consistency of use of clinical components by participants

High rates of variability in dysphagia practice were found in this study, consistent with the results of previous studies (Bateman et al., 2007; Carnaby & Harenberg, 2013; Martino, Pron & Diamant, 2004; Mathers-Schmidt & Kurlinski, 2003). It was interesting that gag reflex testing was used inconsistently given its poor support in the literature (McCullough et al., 2005; Oliveira et al., 2015). It seems that speech-language therapists are not sure whether or not to include this clinical component in the clinical swallow evaluation. Social history and background information may not be used consistently due to time constraints where assessing the swallow is more of a priority. It is, however, surprising that current medication use is inconsistent and may be due to the speech-language therapist thinking that is the doctor’s area of expertise or that the cause of the dysphagia is due solely to the stroke. Poor training at an undergraduate level and at a post-graduate level may also be a factor.

Consistency of clinical practice is desirable; however, it is neither rigid nor prescribed (Carnaby & Harenberg, 2013). It has been known to improve the quality of health care (Carnaby & Harenberg, 2013), and thus adaptations to consistency of practice are endorsed. A high rate of variability in clinical practice is expected in an economically developing country like South Africa where different education and training programmes are established at different universities. Singh et al. (2015) discovered that not only were these education and training programmes varied, but four out of six universities provided inadequate theoretical and clinical training. This leaves half of speech-language therapists feeling unprepared and insufficiently trained in adult dysphagia to perform a clinical swallow evaluation without supervision in the working world (Singh et al., 2015). New speech-language therapists may omit assessing some clinical components in the clinical swallow evaluation due to nervousness or due to lack of theoretical knowledge, training and experience (Singh et al., 2015). Ideally, new graduates should practise under supervision of an experienced colleague for about 6 months (SASLHA, 2011), but often in South Africa this is not possible due to the shortage of staff or due to the fact that new graduates are often the only ones placed at a health institution to increase outreach of health services to the public. The consequences of this include unsatisfactory and potentially unsafe patient management and the speech-language therapist avoiding contributing to the burden of limited access to health care.

Speech-language therapists do not always practise from research-based theory, but rather from experience. Experience results in increased levels of confidence and influences practice. Clinical competency and expertise, and preference and attitude towards practice also play a role in practice patterns (Riquelme, 2015). Practice in South Africa is based on experience at an undergraduate level and in the working world as well as from the opinions of experienced colleagues, thus not being evidence-based (Steele et al., 2007). Barriers to continuing education include time constraints, geographical problems, lack of available courses and financial difficulties (Steele et al., 2007). There can also be a lack of access to articles and research, poor aptitude in identifying information from articles and reduced perceived value and relevance of information found (Nail-Chiwetalu & Ratner, 2007). CANMeds, a framework for medical health professional competency of practice, highlights the importance of life-long learning and continued professional education as well as the translation, distribution and application of learned knowledge (Frank, 2005).

### Resources in the clinical swallowing evaluation

#### Physical resources

More than half of responses indicated poor availability of resources. Speech-language therapists are inclined to make alternative arrangements in evaluation contexts when resources are limited. For example, to adapt food and liquid consistencies without modifying agents by using the South African food *amasi* (fermented milk). This is a liquid with a thicker consistency than milk and is relatively cheap. Speech-language therapists are forced to think ‘out of the box’ and to become practical with little or no resources in the clinical swallow evaluation. Otherwise, infrequent use of resources was logical, and it seems the speech-language therapist uses physical resources sparingly.

The amount of resources used was approximately the same in both public and private health sectors: 42.1% and 57.9%, respectively. However, about 75% of responses mentioning unavailability of physical resources came from participants in the public health sector. Fifty per cent of the participants in the private health sector did not use resources due to unavailability, while the remaining 50% concerned poor training, time limits, patient-specific needs and gaps in protocol. The allocation of financial resources in the public health sector is inadequately managed. Due to poor administrative and managerial capacities and infrastructure the availability of physical resources is often limited (Mayosi et al., 2012). The private health sector receives financial contributions nine times the amount the public health sector receives; hence, there is more money appropriately allocated towards physical resources (Harris et al., 2011). The public–private divide in health sectors in South Africa shows an inequitable and inefficient distribution of resources.

#### Medical resources

Blackwell and Littlejohns (2010) also detected poor usage of medical resources in the clinical swallow evaluation due to the absence thereof. It was mentioned that there is a lack of financial resources specifically for equipment, such as stethoscopes for performing cervical auscultation procedures and oximeters for conducting pulse oximetry procedures. There was a desire from participants to receive further training with the use of such medical resources; however, there may be a lack of funding or support for such training pre- and post-graduation. In the light of a context such as South Africa, it is comforting to see that more than 90% of participants in the study had access to consumable resources *usually* and *always*.

**Non-medical resources:** Infrequent use of food and liquid modifying agents could be due to limited availability and patient-specific needs. Adults with stroke may not require thickeners and often do not like the taste of such products. The fact that their diet is modified at all is alone the cause for a lowered quality of life (Swan, Speyer, Heijnen, Wagg & Cordier, 2015). Infrequent use of adapted eating utensils may have been due to the fact that regular eating utensils are favoured over adaptive feeding tools as they are less expensive and do not stand out in a social eating situation. Some adults with neurogenic dysphagia do not want to be seen in social situations eating with adapted eating utensils. Some participants mentioned trying to aim for typical eating utensils in therapy.

**Human resources:** Multidisciplinary teamwork is key for interprofessional communication and identifying common patient goals (Trapl et al., 2007). There is little regard for other health professionals on the team. Doctors and nurses have been reported to have a poor awareness and regard for speech-language therapists and their role in neurogenic dysphagia (Albini, Soares, Wolf & Goncalves, 2013). One note of concern was that staff changes often caused problems in terms of continuing recommended feeding practices and diets. Speech-language therapists need to document their recommendations extensively and clearly after a clinical swallow evaluation so that other speech-language therapists and other health professionals can continue to engage in correct and safe feeding practices.

There is a drastic shortage of health professionals in South Africa compared with high-income countries, especially in the public health sector and in rural areas (George et al., 2009; Mills et al., 2011). For every patient seen by a specialist in the private health sector 23 are seen by a specialist in the public health sector (George et al., 2009). Reasons for such human resource shortages include migration of health professionals, the ageing of the nurse population and the increasing burden of disease and illness (George et al., 2009). The extension of the national antiretroviral treatment programme for HIV and AIDS has also drawn nurses away from other health services (George et al., 2009).

It is evident that access to resources is most often a challenge in a context like South Africa. Reasons for poor resource use in the clinical swallow evaluation are indicative of South Africa’s poor resource availability and insufficient training. The speech-language therapist therefore uses certain resources less frequently and needs to adapt the clinical swallow evaluation to suit the limited availability of resources and the needs of the adult with acute stroke.

### Limitations

As clinical practice represents one’s ability to manage a patient optimally, participants may have been more likely to report higher frequencies of clinical component use due to these relatively sensitive questions. This observational error could have occurred due to knowledge of scope of practice and service expectations and to avoid embarrassment (Groves, 2004). The sample selection methods may have been biased due to using only one organisation to recruit participants. This may have obtained an unrepresentative sample of the population, as participants did not have an equal chance of being selected for the study (Leedy & Ormrod, 2013). A bigger sample size could have been obtained; however, due to the nature of electronic surveys, response rates are often low (Cook, Heath & Thompson, 2000). Generalisation of results must be considered with caution and observations can only be made with regard to the participants in this study. The qualitative data component was subject to theme identification by the researcher only, of which the reliability has been known to be poor. It is however widely accepted for researchers to review their own data, especially as it is not a mixed method study (Carnaby & Harenberg, 2013; Mathers-Schmidt & Kurlinski, 2003).

### Validity and reliability

The researcher conducted a narrative review of the literature to adapt the already existing questionnaire from other validated studies (Green et al., 2006). The reliability of the narrative review was increased by having external reviewers blindly review databases (Leedy & Ormrod, 2013). One hundred per cent inter-rater agreement was reached for any disparities between reviewers. Construct validity was improved by keeping search terms consistent among reviewers and by selecting reviewers who were also speech-language therapists thus having an adequate understanding of the research topic and search terms (Leedy & Ormrod, 2013).

A pilot study was conducted to identify any weaknesses in the questionnaire’s content and layout and with regard to its applicability in the South African context (Leedy & Ormrod, 2013). This improved internal consistency reliability and construct validity (Leedy & Ormrod, 2013). By having South African participants take part in the pilot study the terminology could be verified as appropriate or not and contextual factors influencing questions or answers could be commented on.

Developing a questionnaire that was similar to, motivated from and adapted from other, recent and validated studies increased the content validity and criterion-related validity (Leedy & Ormrod, 2013). Reliability was also improved by eliminating interview bias by administering the questionnaire by only electronic means (Leedy & Ormrod, 2013). The questionnaire was worded and presented clearly and unambiguously, and without misleading tendencies or persuasion (Babbie & Mouton, 2001) to ensure a greater likelihood of truthful and relevant answers.

## Ethical considerations

Ethical permission from the Biomedical Research Ethics Committee at the University of KwaZulu-Natal was granted in April 2015. Participation in the study was voluntary and the participant could withdraw from the study at any time. Consent was indicated by their signing the consent form, and all responses were kept confidential and anonymous (Leedy & Ormrod, 2013). Participants were not subjected to any physical, psychological or disclosure dangers and were not obliged to participate. Participants benefitted only by receiving feedback of the study’s results. Copies of the completed questionnaires were kept confidential and will be stored electronically at the University of KwaZulu-Natal for up to 5 years post-completion of the study and thereafter digitally destroyed with two staff members who will bear witness to the procedure. Arbitrary numbers were allocated to each completed questionnaire and raw data responses were shared with only the statistician (Leedy & Ormrod, 2013).

## Conclusion

Varying adherence to official policies and fluctuating consistency of clinical practice among speech-language therapists in this study indicate a need for more uniformity of education and training curricula at South African universities as well as for more supervision after graduation. It is also apparent that continued professional development is important for updated knowledge and practice. A means by which speech-language therapists can access recent literature is vital in order to maintain knowledge and engage in evidence-based practice. Policies set out to guide clinical practice need perhaps to be more detailed, more easily available and more recognised. A suitable clinical swallowing evaluation policy specifically for a resource-limited context is also needed, and more human, financial and physical resources are also essential. Ultimately, the adult with neurogenic dysphagia post-acute stroke may not benefit fully from such a context and from inconsistent clinical practice in South Africa.

More research is needed with regard to the frequency of clinical component use, the consistency of its use and reasons why it is not used in the clinical swallow evaluation of adults with neurogenic dysphagia. Reviews of what current curricula entail at South African universities are needed to determine how many hours of theoretical and practical training are completed and which clinical components are being taught. Current policies and guidelines also need to be reviewed to address uniformity and comprehensiveness among them. A suitable clinical swallowing evaluation policy for a resource-limited context is also needed. More research is needed potentially to motivate for an improved curriculum at an educational level through which a more consistent clinical swallow evaluation protocol with a constant set of clinical components is taught at universities across South Africa. This is in the hope that the use and consistency of clinical components by speech-language therapists in the clinical swallowing evaluation of adults with neurogenic dysphagia post-acute stroke will improve.
